# Genome-Wide Identification and Functional Prediction of Long Non-coding RNAs Involved in the Heat Stress Response in *Metarhizium robertsii*

**DOI:** 10.3389/fmicb.2019.02336

**Published:** 2019-10-09

**Authors:** Zhangxun Wang, Yuanyuan Jiang, Hao Wu, Xiangyun Xie, Bo Huang

**Affiliations:** ^1^Anhui Provincial Key Laboratory of Microbial Pest Control, Anhui Agricultural University, Hefei, China; ^2^School of Plant Protection, Anhui Agricultural University, Hefei, China

**Keywords:** *Metarhizium robertsii*, long non-coding RNAs, heat stress, RNA-seq, transcriptional profiling

## Abstract

Long non-coding RNAs (lncRNAs) play a significant role in stress responses. To date, only a few studies have reported the role of lncRNAs in insect-pathogenic fungi. Here, we report a genome-wide transcriptional analysis of lncRNAs produced in response to heat stress in *Metarhizium robertsii*, a model insect-pathogenic fungus, using strand-specific RNA sequencing. A total of 1655 lncRNAs with 1742 isoforms were identified, of which 1081 differentially expressed (DE) lncRNAs were characterized as being heat responsive. By characterizing their genomic structures and expression patterns, we found that the lncRNAs possessed shorter transcripts, fewer exons, and lower expression levels than the protein-coding genes in *M. robertsii*. Furthermore, target prediction analysis of the lncRNAs revealed thousands of potential DE lncRNA–messenger RNA (mRNA) pairs, among which 5381 pairs function in the *cis*-regulatory mode. Further pathway enrichment analysis of the corresponding *cis*-regulated target genes showed that the targets were significantly enriched in the following biological pathways: the Hippo signaling pathway and cell cycle. This finding suggested that these DE lncRNAs control the expression of their corresponding neighboring genes primarily through environmental information processing and cellular processes. Moreover, only 26 *trans*-regulated lncRNA–mRNA pairs were determined. In addition, among the targets of heat-responsive lncRNAs, two classic genes that may be involved in the response to heat stress were also identified, including *hsp70* (XM_007821830 and XM_007825705). These findings expand our knowledge of lncRNAs as important regulators of the response to heat stress in filamentous fungi, including *M. robertsii*.

## Introduction

*Metarhizium robertsii*, a model insect-pathogenic fungus, has been widely used for biological pest control (Fang et al., 2012; Wang and Wang, 2017). In addition to its virulence, the sensitivity of *Metarhizium* to environmental stress, including high temperature and ultraviolet (UV) rays, is the main obstacle for its use as a reliable pest control agent (Lovett and St Leger, 2015, 2018). For example, its conidial viability has been reported to be severely reduced by high temperature in the field (Rangel et al., 2006; Lovett and St Leger, 2015). Therefore, for commercial development and genetic improvement of *Metarhizium* spp., more knowledge about the environmental stress response process in this fungus is needed (Lovett and St Leger, 2015, 2018). Recently, great progress has been made in the recognition and functional identification of protein-coding genes in *Metarhizium* involved in the response to environmental stress, especially heat stress (Liao et al., 2014; Wang et al., 2014; Zhang et al., 2017). However, there are only a few reports regarding the roles of long non-coding RNAs (lncRNAs) in insect-pathogenic fungi.

Long non-coding RNAs, a representative class of transcribed non-coding RNAs, are transcripts with >200 nucleotides (nt) (Ma et al., 2013). Similar to mRNAs, many lncRNAs with a 5′ cap and poly-A tail are transcribed and spliced from their corresponding genes; however, not all lncRNAs display these characteristics (Mercer et al., 2009; Quinn and Chang, 2016). LncRNAs are usually transcribed by RNA polymerase II and RNA polymerase III at several sites of the genome in eukaryotes (Mercer et al., 2009; Quinn and Chang, 2016). In addition, lncRNAs are poorly conserved among different species and are usually expressed at low levels (Mercer et al., 2009; Ma et al., 2013). LncRNAs can be divided into four subcategories according to their genomic location and context: intergenic lncRNAs, antisense lncRNAs, sense lncRNAs, and intronic lncRNAs (Ma et al., 2013; Ransohoff et al., 2018). LncRNAs can regulate the expression of their corresponding target genes in *cis*- and *trans*-acting modes (Kopp and Mendell, 2018) and thus play a significant role in a wide range of biological processes (Mercer et al., 2009; Ponting et al., 2009), such as transcription, translation, the cell cycle, and heat shock response (Shamovsky et al., 2006).

A recent report has shown that lncRNAs play an essential role in the cellular response to different stressful conditions, such as heat stress (Valadkhan and Valencia-Hipolito, 2016). For example, one of the earliest reports regarding the involvement of lncRNAs in the heat stress response focused on a non-coding RNA (heat shock RNA-1, HSR1) that interacted with a translation elongation factor (eEF1A) and controlled the activation of heat shock transcription factor 1 (HSF1) following heat shock (Shamovsky et al., 2006). Recently, an increasing number of lncRNAs related to heat stress responses have been reported in plants (Zhao et al., 2016; Sun et al., 2018). For instance, hundreds of stress-responsive lncRNAs from *Arabidopsis thaliana* have been identified and characterized following treatment with various stress stimuli (including heat conditions) (Di et al., 2014). In recent years, the number of lncRNAs identified in yeast has increased rapidly (Till et al., 2018a); in particular, an increasing number of stress-responsive lncRNAs have been found in yeast (Sun et al., 2019). Furthermore, several recent studies have examined the lncRNA-associated growth and development of filamentous fungi such as *Neurospora crassa* (Arthanari et al., 2014; Cemel et al., 2017), *Fusarium graminearum* (Kim et al., 2018), *Trichoderma reesei* (Till et al., 2018b), and *Cordyceps militaris* (Wang y. et al., 2019). However, there are only a few comprehensive surveys regarding the lncRNAs involved in the response to heat stress in filamentous fungi, including *M. robertsii*.

Here, strand-specific RNA sequencing (RNA-seq) was performed to analyze the genome-wide transcriptomic changes in *M. robertsii* conidia following treatment with high temperatures. Subsequently, high temperature-responsive lncRNAs were systematically identified and functionally predicted. These findings will provide a basis for expanding our knowledge of the lncRNAs involved in high-temperature stress responses in filamentous fungi.

## Materials and Methods

### Fungal Culture and Sample Preparation

*Metarhizium robertsii* strain ARSEF 2575 was cultured on potato dextrose agar (PDA, 20% potato, 2% dextrose, and 2% agar, w/v) plates at 28°C for 14 days to obtain the conidia. The conidia were suspended in a sterile aqueous solution of 0.05% Tween 80, and the conidial suspension was filtered to remove the hyphal debris. Conidial germination assays were performed based on the relative germination of the conidia, as described in our previous study (Wang et al., 2014; Wang Z.X. et al., 2019). Briefly, 1-ml aliquots of the conidial suspensions were collected in 1.5-ml Eppendorf tubes and incubated in a water bath at 40°C (heat treatment) or 28°C (control) for up to 4 h. After heat treatment, 10 μl of the conidial suspension from each tube was dropped onto the centers of PDA plates. Subsequently, the conidial germination percentage on each plate was observed and assessed by means of microscopic counts after 24 h of incubation. There were at least 300 conidia per plate for evaluation; the conidia showing visible germ tubes were considered to have germinated (Wang et al., 2014; Wang Z.X. et al., 2019). Each treatment was conducted with three independent trials. All samples were harvested and subjected to total RNA extraction or stored in liquid nitrogen for subsequent use.

### Total RNA Extraction, Library Preparation, and Sequencing

Total RNA, library construction, and strand-specific RNA-seq were conducted by the Beijing Genomics Institute (BGI, Shenzhen, China). Total RNA was extracted using TRIzol Reagent (Invitrogen, Carlsbad, United States) following the manufacturer’s instructions. The total RNA was examined using a Nanodrop ND-2000 spectrophotometer (Thermo Scientific, Waltham, MA, United States) and an Agilent 2100 Bioanalyzer (Agilent Technologies, Santa Clara, CA, United States). Total RNA (500 ng) from the heat-treated or control conidial samples (three biological replicates for each treatment: a total of six samples) was prepared for RNA-seq library construction. The libraries were constructed using the Illumina TruSeq^TM^ RNA Sample Prep Kit (Illumina, San Diego, CA, United States), along with the Ribo-Zero^TM^ rRNA Removal Kit (Epicentre, Madison, United States) for the removal of ribosomal RNA (rRNA), according to the manufacturer’s recommendations, and sequenced on an Illumina Hiseq X Ten system for generating 150-bp paired-end reads. The data were deposited in the National Center for Biotechnology Information database under the accession number PRJNA232660.

### LncRNA Identification and Annotation

Clean reads were acquired by removing the reads mapped to rRNAs and trimming raw reads that had unknown, low-quality, or adaptor sequences and were mapped to the *M. robertsii* genome using HISAT (hierarchical indexing for spliced alignment of transcripts), allowing up to two read mismatches and read gap length (Gao et al., 2011; Kim et al., 2015). Subsequently, the transcripts were assembled using StringTie software (Pertea et al., 2015) and the available *M. robertsii* annotation as the reference (Gao et al., 2011). After reconstruction analysis of the transcripts, the corresponding transcripts were compared to known gene models by Cuffcompare [one of the tools for Cufflinks (Trapnell et al., 2010)], and the corresponding information of their location relationships was obtained. For identification of lncRNAs in *M. robertsii*, several steps were used, as described in a previous study (Wang Y. et al., 2018; Wang Z. et al., 2018): (1) The initial transcripts were compared to known genes using Cuffcompare software (Trapnell et al., 2010), and only transcripts at the non-gene loci were selected for further analysis. (2) The assembled transcripts with length < 200 nt and those with open reading frame (ORF) length > 100 amino acids (aa) were excluded. (3) The assembled transcripts with coding potential (potential ORFs) were removed according to the evaluation of the Coding Potential Calculator (CPC) (Kong et al., 2007), txCdsPredict, and Coding-Non-Coding Index (CNCI) (Sun et al., 2013). In addition, the assembled transcripts, including known protein domains, were also removed, following their comparison with sequences in the Pfam protein family database (Finn et al., 2016). (4) The corresponding transcripts with a certain level of transcription were considered lncRNA candidates with high confidence.

Furthermore, for quantification of the lncRNAs, their expression levels were determined as fragments per kilobase of transcript per million mapped reads (FPKM) (Trapnell et al., 2012). The assembled transcripts with FPKM value > 1 in at least one RNA-seq library were considered as lncRNAs for subsequent analysis. Differential expression was determined using a false discovery rate (FDR) of less than 0.001 and an absolute value of the log_2_ (fold change) of more than 1 as the threshold, as mentioned in a previous study (Anders and Huber, 2010; Wang et al., 2010).

### Prediction of the Target Genes of lncRNAs and Functional Enrichment Analysis

Long non-coding RNAs mainly control the expression of corresponding target genes in a *cis-* or *trans*-acting manner (Rinn and Chang, 2012; Quinn and Chang, 2016; Kopp and Mendell, 2018). For identification of the *cis*-regulatory lncRNAs and their target genes, lncRNAs were deemed to be *cis*-regulatory if they were located within 10/20 kb upstream/downstream of the corresponding mRNA. When lncRNA–mRNA pairs span beyond this range in the genomic distance, the corresponding lncRNAs and their potential target genes were further predicted by the RNAplex tool (Tafer and Hofacker, 2008), and the binding free energy was evaluated. If the minimum free energy was <−30 kcal/mol, the corresponding lncRNA was identified as a *trans*-regulatory lncRNA (Wang Z. et al., 2018).

To investigate the potential function of these lncRNAs in *M. robertsii*, Kyoto Encyclopedia of Genes and Genomes (KEGG) pathway enrichment analysis for differentially expressed (DE) target protein-coding genes of the lncRNAs was performed using the information in the KEGG pathway database (Kanehisa et al., 2012). A hypergeometric test was performed to determine the significantly enriched KEGG pathways, and the significantly enriched KEGG pathways were identified using an adjusted *p*-value of 0.05 as a threshold, as reported in a previous study (Wang Z.X. et al., 2018). Moreover, the interacting networks between DE lncRNAs and their target mRNAs in the significantly enriched KEGG pathways were visualized using Cytoscape software (Smoot et al., 2011).

### RT-PCR Assays

To validate the results of strand-specific RNA-seq analysis for the corresponding selected lncRNAs, semiquantitative RT-PCR assays were performed as described previously (Donaldson and Saville, 2013; Wang Y. et al., 2018), and *gpd* (glyceraldehyde 3-phosphate dehydrogenase, MAA_07675) was used as an internal control (Fang and Bidochka, 2006). The extraction and examination of the total RNA were conducted as mentioned previously, and the concentration of RNA for all the different samples was adjusted to 100 ng μl^–1^. First-strand cDNA was synthesized from 1 μl of total RNA using the PrimeScript^TM^ II 1st Strand cDNA Synthesis Kit (TaKaRa, Dalian, China); the RT reactions were performed using the RT Primer Mix (oligo-dT and random hexamers). The synthesized cDNA (10× dilution) was used as the template for the subsequent PCR amplification. PCR amplification was performed using the 2× Taq Master Mix (Dye Plus) (Vazyme, Nanjing, China) according to the manufacturer’s instructions. The conditions for the amplification were as follows: denaturation at 95°C for 3 min, followed by 30 cycles of 30 s at 95°C, 30 s at 54°C, and 30 s at 72°C, followed by extension at 72°C for 5 min. All the primers used are listed in Supplementary Table S1.

### Gene Deletion and Complementation

Deletion of the lncRNA gene (Gene_ID: LXLOC_012667, Transcript_ID: TCONS_00017175, referred to herein as *mrLncRNA1*) was performed by introducing a glufosinate ammonium (bar) resistance marker in the lncRNA gene of interest by *Agrobacterium*-mediated fungal transformation as described previously (Chacko et al., 2015; Wang y. et al., 2019; Wang Z.X. et al., 2019). Briefly, the 5′-flanking region (1068 bp) and 3′-flanking region (1043 bp) of the lncRNA gene (*mrLncRNA1*) were amplified from genomic DNA using high-fidelity Taq DNA polymerase (KOD Plus Neo, Toyobo, Osaka, Japan) (Supplementary Figure S1A) and inserted into the binary vector pDHt-SK-bar (conferring resistance to glufosinate ammonium) for fungal transformation to generate the gene deletion mutant Δ*mrLncRNA1*. For complementation, the *mrLncRNA1* transcript together with its promoter region was amplified by PCR, and the product was digested with *Spe*I and inserted into the binary vector pDHt-SK-*ben* (conferring resistance to benomyl) for ectopic integration into Δ*mrLncRNA1* to obtain the complemented strain (Comp). The positive transformants were confirmed by PCR and RT-PCR as previously described (Wang y. et al., 2019; Wang Z.X. et al., 2019) (Supplementary Figures S1B,C). Primers used for gene deletion and complementation analysis are shown in Supplementary Table S1. In the heat stress assay, 1-ml aliquots of conidial suspensions in 1.5-mL Eppendorf tubes were incubated in a water bath at 40°C for 90 min (Wang Z.X. et al., 2019). Subsequently, 10-μl aliquots of the treated suspensions were spread onto PDA plates and incubated at 25°C for 16 h and 24 h. The percentage of conidial germination and relative germination rate were assessed as described above.

## Results

### Effects of Heat on Conidial Germination

For exploring heat stress responses in *M. robertsii*, the relative germination rate of *M. robertsii* conidia at different temperatures was evaluated to identify an optimal temperature for the functional genomics analysis; previously, we used a temperature of 38°C as the treatment temperature for transcriptomic analysis (Wang et al., 2014). Our results showed thousands of DE genes between the heat-treated and control conidial samples (Wang et al., 2014). However, according to a recent report, no DE lncRNAs were discovered in *N. crassa* following treatment at a temperature of 30°C for 1 h (Arthanari et al., 2014). In this study, we found that the viability of the conidia was approximately 75% (in terms of relative percent germination) following treatment at 40°C for 4 h; this finding suggested that the heat stress conditions obtained following treatment of the conidia at a temperature of 40°C can be used for functional genomics analysis, as described in a previous report (Rangel et al., 2006; Wang et al., 2014; Sun et al., 2019). Therefore, in this study, to maximize the coverage of the heat-responsive lncRNAs in *M. robertsii*, we treated the conidial samples at a temperature of 40°C for 4 h for the lncRNA transcriptomic analysis. Subsequently, strand-specific RNA-seq for the heat-treated (exposed to a temperature of 40°C) and control (28°C) conidial samples was performed for systematic analysis of heat-responsive lncRNAs in *M. robertsii*.

### Identification and Characterization of lncRNAs From *M. robertsii*

**FIGURE 1:**
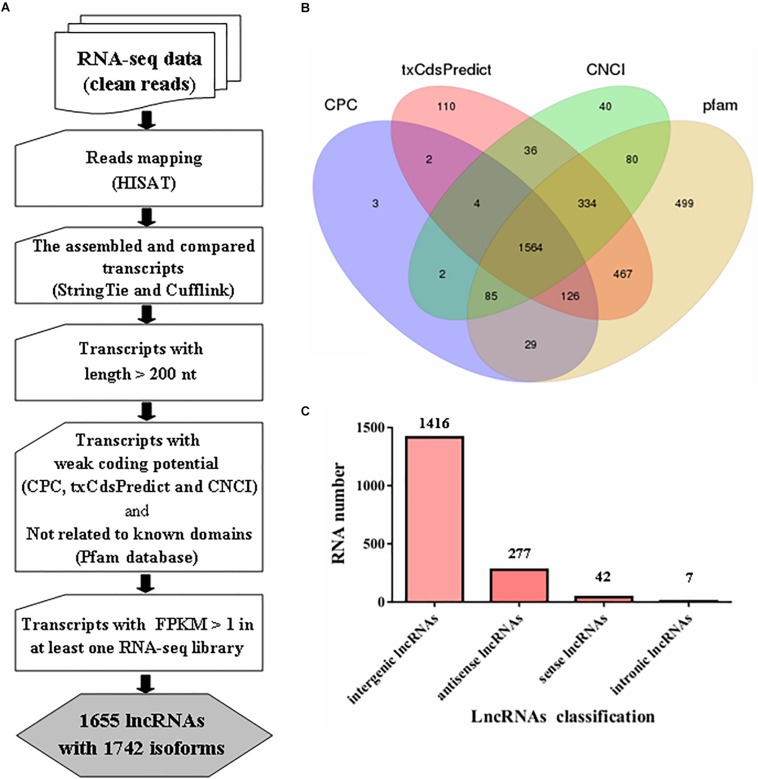
Prediction of lncRNAs in *Metarhizium robertsii*. **(A)** Flowchart for the analysis of lncRNAs in *M. robertsii*. **(B)** Venn diagram analysis of the numbers of candidate lncRNAs filtered by CPC, txCdsPredict, CNCI, and comparison with the Pfam database. **(C)** Number of lncRNAs in different categories.

In our analysis, approximately 28.5 Gb of data were obtained from six libraries after removing the contaminated and low-quality reads; approximately 75.91% of the clean reads were mapped to the reference genome of *M. robertsii* for further analysis (Supplementary Table S2). Subsequently, systematic analysis of lncRNAs in *M. robertsii* was performed based on strand-specific RNA-seq data (Figure 1A).

For identification of lncRNAs in *M. robertsii*, a total of 14,517 transcripts were obtained after reconstruction analysis of the transcriptome obtained from the strand-specific RNA-seq data in six libraries. Based on the assembled 14,517 transcripts, several steps were implemented to identify the heat-related lncRNAs in *M. robertsii* (Figure 1A). First, all the transcripts were compared to the gene annotation data using Cuffcompare software; this process generated different transcripts at the non-gene loci containing candidate lncRNAs, which were chosen based on the following criteria: transcripts with >200 nt and transcripts with a potential ORF with <100 aa. Second, different programs, including CPC, txCdsPredict, and CNCI, were used to analyze the coding potentials of the corresponding transcripts, and only those with a weak coding potentials (CPC score < 0, txCdsPredict score < 500, and CNCI score < 0) were retained. In addition, the transcripts were also compared to sequences in the Pfam protein family database to remove the corresponding transcripts that may encode conserved domains (Figure 1B). Third, the corresponding transcripts with a certain level of transcription (FPKM value > 1 at least in one RNA-seq library) were identified as lncRNAs with high confidence. Finally, we identified 1655 lncRNAs with 1742 isoforms, of which 1416 were classified as intergenic lncRNAs (also known as lincRNAs, 1416/1742 = 81.28%), 277 were classified as antisense lncRNAs (15.9%), 42 were classified as sense lncRNAs, and seven were classified as intronic lncRNAs (Figure 1C and Supplementary Table S3).

**FIGURE 2:**
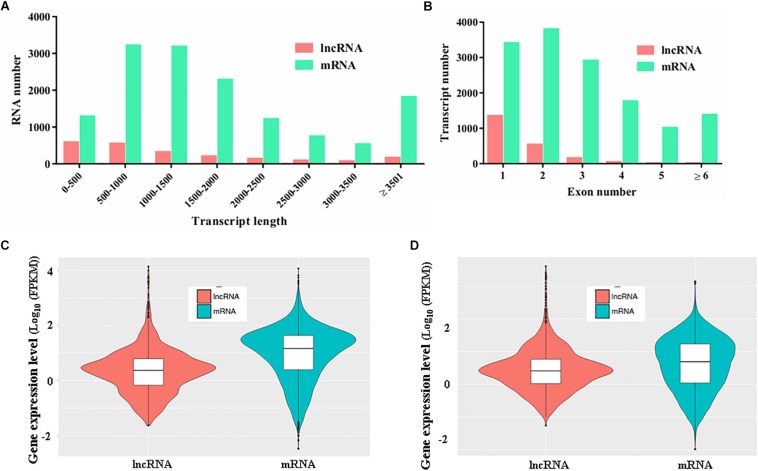
Characteristics of the predicted novel lncRNAs in *M. robertsii*. **(A)** Transcript lengths of protein-coding genes and lncRNAs. **(B)** Number of exons per transcript for protein-coding genes and lncRNAs. **(C)** Gene expression levels of protein-coding genes and lncRNAs in the control conidial samples. **(D)** Gene expression levels of protein-coding genes and lncRNAs in heat-treated conidial samples.

To characterize the features of the identified lncRNAs in *M. robertsii*, the lengths of the transcripts, number of exons, and gene expression levels of the lncRNAs were assessed and compared to those of the protein-coding genes. Our results showed that there were obvious differences between the protein-coding genes and lncRNAs with regard to the distribution of transcript length and exon number. For instance, the transcripts of lncRNAs in *M. robertsii* were markedly shorter than those of the protein-coding genes, and most of the *M. robertsii* lncRNAs (approximately 68.72%) were shorter than 1500 nt (Figure 2A). Moreover, approximately 89.32% (1556 out of 1742) of the lncRNAs contained no more than two exons; this statistic was >50.45% in the case of the protein-coding genes (Figure 2B). In addition, the expression level of lncRNAs was notably lower than that of the protein-coding genes, according to the calculation of the FPKM value for each transcript, regardless of whether the conidial samples were heat treated (40°C) or exposed to a temperature of 28°C (control) (Figures 2C,D). Overall, our results provide a transcriptional overview of the lncRNA changes in *M. robertsii* in response to heat stress.

### LncRNAs Showing Differential Expression Under Heat Stress Conditions

To investigate the changes in lncRNA transcription under conditions of heat stress, DE lncRNAs were identified based on the following criteria: absolute value of the log_2_ (fold change) ≥ 1 and FDR ≤ 0.001. In total, 1081 DE lncRNAs were identified (Supplementary Table S4). Of these lncRNAs, 726 were found to be upregulated and 355 were found to be downregulated under heat stress conditions (Supplementary Table S4). Among the DE lncRNA transcripts, LTCONS_00012285 was found to be the most upregulated, with a fold change of 499.95, whereas LTCONS_00016143 was found to be the most downregulated, with a fold change of 538.55, after comparison of the heat-treated samples with the control samples (Supplementary Tables S3, S4).

**FIGURE 3:**
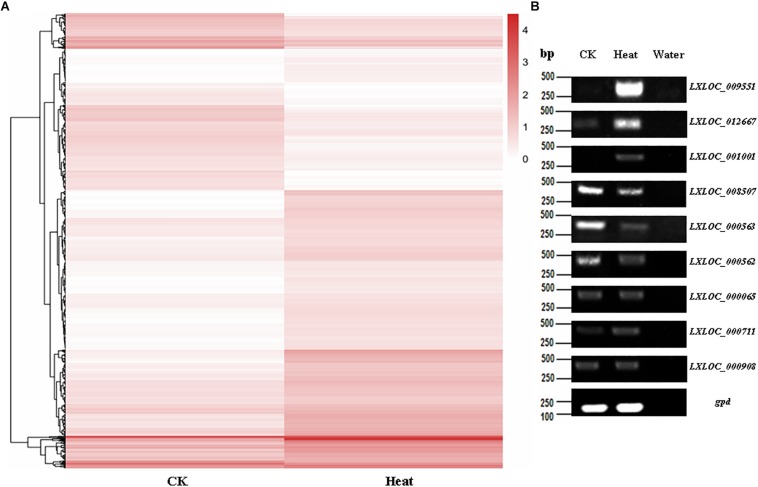
Transcription patterns of lncRNAs in *M. robertsii*. **(A)** Heatmaps showing the DE lncRNAs in the heat-treated and control conidial samples. Transcription patterns of the lncRNAs in the heat-treated and normal conidia samples were obtained by hierarchical clustering, and normalization of log_10_ (FPKM) was used to determine the gene expression levels in different samples. **(B)** Semiquantitative RT-PCR assay to confirm the expression of the corresponding lncRNAs.

Furthermore, heatmaps were also produced by hierarchical clustering analysis, and the DE lncRNAs were distinctly self-isolated into clusters (Figure 3A). Moreover, to further verify the results of the lncRNA analysis, nine DE lncRNAs among the heat-treated and control conidial samples were chosen for semiquantitative RT-PCR, using *gpd* (MAA_07675) as an internal control. Our results showed that the lncRNAs that showed relatively high expression levels in the RNA-seq analysis of the heat-treated (*LXLOC_009551*, *LXLOC_012667*, and *LXLOC_001001*) and control (*LXLOC_008507*, *LXLOC_000562*, and *LXLOC_000563*) conidial samples also displayed brighter bands in the semiquantitative RT-PCR analysis of the corresponding samples (Figure 3B). Additionally, three lncRNAs (*LXLOC_000065*, *LXLOC_000711*, and *LXLOC_000908*) that showed no changes in their transcription levels in the RNA-seq analysis yielded bands of similar brightness after PCR amplification of the heat-treated and untreated conidial samples (Figure 3B). Overall, the results of the semiquantitative RT-PCR analysis were consistent with those of the RNA-seq data analysis.

### Functional Analysis of lncRNAs Showing Differential Expression Under Heat Stress Conditions

**FIGURE 4:**
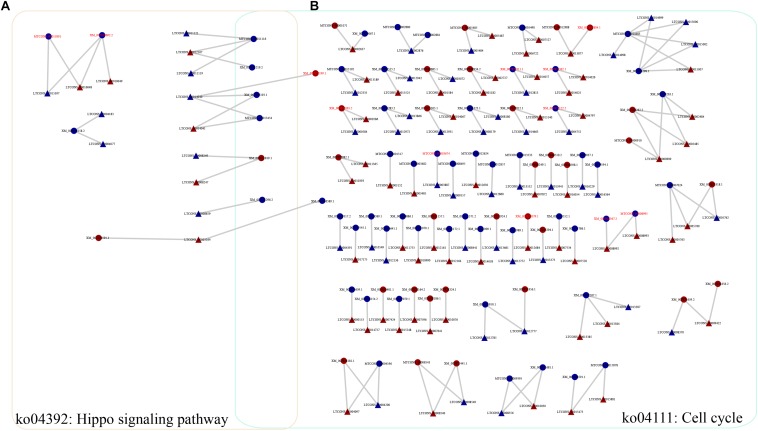
Representative relationships for the network of lncRNAs and their *cis*-regulated target genes. **(A)** Network of lncRNAs with their *cis*-regulated target genes in the Hippo signaling pathway. **(B)** Network of lncRNAs with their *cis*-regulated target genes in the cell cycle pathway. Triangles and circles represent lncRNAs and their target genes, respectively. Red font indicates that the target gene is a transcription factor. Red, upregulated; blue, downregulated.

To investigate the functions of heat-responsive lncRNAs, first, the potential *cis*-regulated targets of the DE lncRNAs were predicted using 1081 DE lncRNAs and 7003 DE genes. In total, 5381 *cis*-regulatory lncRNA–mRNA pairs were identified by this analysis, Of which, lncRNAs in 201 cis-regulatory lncRNA–mRNA pairs were overlapped with target mRNAs, whereas lncRNAs in 5180 cis-regulatory lncRNA–mRNA pairs were localized within 10 k/20 kb upstream and downstream of the nearby DE target genes (Supplementary Table S5). To further reveal the biological pathway information for the potential targets of these heat-responsive DE lncRNAs, the corresponding potential target genes were subjected to KEGG pathway analysis. Our results showed that these DE target genes could be mapped to 126 pathways. Among these different pathways, nine significantly enriched KEGG pathways were determined, with at least four related DE target genes (Supplementary Table S6), when the *p*-value was adjusted to 0.05. The Hippo signaling pathway (multiple species) and cell cycle (yeast) were the most and second most enriched pathways, respectively (Figure 4 and Supplementary Table S6). Other representative pathways also included fatty acid metabolism, carbon metabolism, and biosynthesis of unsaturated fatty acids (Supplementary Table S6). Altogether, our results indicated that these lncRNAs, which may function as *cis*-regulatory lncRNAs in response to heat stress, controlled the expression levels of their neighboring genes primarily by environmental information processing (signal transduction), cellular processes, and other metabolic processes.

In addition, only 26 *trans*-acting lncRNA–mRNA pairs were identified in our study (Supplementary Figure S2 and Supplementary Table S7). In particular, several lncRNAs and target genes showed increased expression under heat stress relative to their expression under control conditions (lncRNA–mRNA pairs for P4, P5, P6, P8, P12, and P26 in Supplementary Figure S2 and Supplementary Table S7). For example, target genes (XM_011412672) of the lncRNA (LTCONS_00008993) in P12 encoded a C2H2 zinc finger transcription factor, and target genes (XM_007828519) of the lncRNA (LTCONS_00017373) in P26 encoded a zinc knuckle; these results indicated that these lncRNAs may be involved in the regulation of transcription. Moreover, several upregulated lncRNAs and their corresponding target genes, the expression of which was downregulated following heat stress, were also identified (lncRNA–mRNA pairs for P11, P13, P15, P16, and P24 in Supplementary Figure S2 and Supplementary Table S7). These corresponding target genes encoded novel transcripts or hypothetical proteins, which needs to be clarified in further studies.

Moreover, for the most upregulated lncRNA (LTCONS_00012285), the results of target gene prediction show that two target genes (XM_007828495.2 and XM_007828496.1 in Supplementary Table S5) were found to function in the cis-regulatory mode, and the expression of these two target genes was significantly decreased under heat stress conditions. In addition, for the most downregulated lncRNA (LTCONS_00016143), the corresponding target genes were also predicted, and the results show that there are four target genes (Supplementary Table S5). The expression of two target genes [XM_007818914.1 (encoding a zinc finger transcription factor) and XM_011413457.1] was markedly increased after heat treatment (Supplementary Table S5).

### Potential Function of *mrLncRNA1* in Conidial Thermotolerance

The *mrLncRNA1* was selected for analysis of its potential function in conidial thermotolerance by targeted gene deletion based on homologous recombination. In our study, the expression level of *mrLncRNA1* in heat-treated conidia was 218.63 times higher than that in control samples; that is, the expression level of *mrLncRNA1* was significantly increased under heat treatment conditions.

**FIGURE 5:**
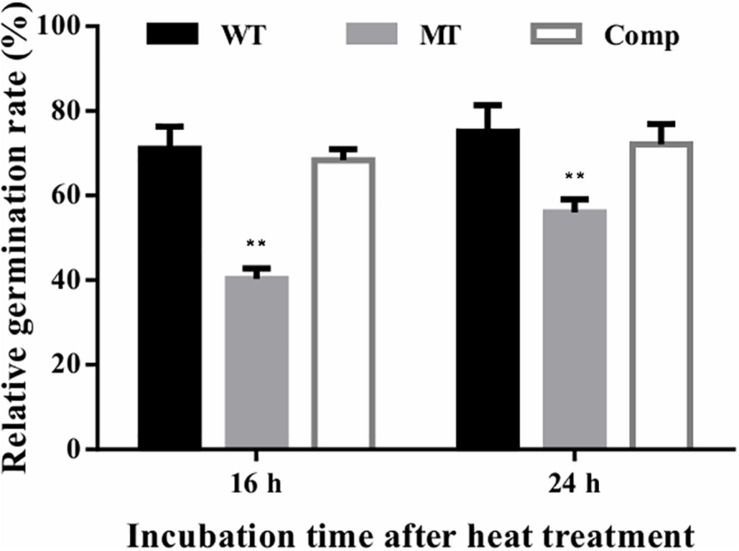
Conidial tolerance to heat treatment. Relative germination percentage of conidia at 16 and 24 h of incubation after exposure to heat treatment. Germination rates were determined after inoculation on PDA at 25°C for 16 and 24 h, and relative germination percentage was calculated with respect to the untreated control. ^∗∗^*p* < 0.01. WT, wild-type strain; MT, Δ*mrLncRNA1* strain; Comp, complemented strain.

The effect of lncRNA gene deletion on conidial tolerance to heat shock was assessed based on the relative germination rates of stressed conidia versus unstressed control conidia after 16 or 24 h of incubation. In the present study, the relative germination rates of the lncRNA gene deletion strain (Δ*mrLncRNA1*) were lowered by 31 and 19% at 16 and 24 h after incubation, respectively, compared to that of the control strain (Figure 5). These results suggest that *mrLncRNA1* contribute significantly to conidial thermotolerance in *M. robertsii*.

## Discussion

An increasing number of studies have shown that lncRNAs are widely present in eukaryotes (Ponting et al., 2009; Quinn and Chang, 2016; Till et al., 2018a). LncRNAs play different essential roles in various organisms, including fungi (Quinn and Chang, 2016; Valadkhan and Valencia-Hipolito, 2016; Sun et al., 2018; Till et al., 2018a). However, a comprehensive analysis of the profiles of lncRNAs showing differential expression under heat stress conditions in filamentous fungi has not yet been reported. To explore the expression profiles and functions of lncRNAs from *M. robertsii* that are involved in the response to heat stress, we performed a genome-wide analysis of lncRNAs in heat stress-treated *M. robertsii* conidial samples by strand-specific RNA-seq. We finally identified 1655 lncRNAs with 1742 isoforms, of which 1416 were classified as lincRNAs. Our results for the distribution patterns of the different lncRNA classes were consistent with those of previous studies (Supplementary Table S8), which have stated that a majority of fungal lncRNAs are located in intergenic regions (Ma et al., 2013; Kyriakou et al., 2016; Till et al., 2018a). Further analysis showed that most *M. robertsii* lncRNAs (89.32%) have 1–2 exons; these lncRNAs had features similar to those reported in previous studies (Ma et al., 2013; Wang Y. et al., 2018).

Previously, lncRNAs have also been reported to participate in regulating the response to abiotic stress, including heat stress (Valadkhan and Valencia-Hipolito, 2016). In our analysis, 1081 DE lncRNAs were identified; 726 lncRNAs were found to be upregulated and 355 lncRNAs were found to be downregulated under heat-treatment conditions. It has been reported that lncRNAs can have significant effects on transcriptional regulation via either *cis*- or *trans*-acting modes and can thus positively or negatively regulate the expression of protein-coding genes. Of these lncRNAs, *cis*-regulatory lncRNAs mainly control the expression of neighboring genes (close genomic proximity), and *trans*-regulatory lncRNAs modulate the expression of distant genes (Rinn and Chang, 2012; Quinn and Chang, 2016). Therefore, to further reveal the potential function of lncRNAs, the putative *cis*- or *trans*-regulated targets of lncRNAs were analyzed. In our analysis, we selected only those pairs in which both the lncRNA and its protein-coding gene showed differential expression under heat stress conditions. These 7003 DE genes in the heat-treated and control conidial samples were further subjected to functional prediction as the targets of lncRNAs; KEGG pathway annotations were obtained for these genes according to rich factor analysis (Wang Z.X. et al., 2018). Our results showed that the altered mRNAs were mainly enriched in different pathways, including carbon metabolism, fatty acid metabolism, ribosome, pyruvate metabolism, and cell cycle (Supplementary Figure S3). Collectively, these findings are consistent with those from previous studies by our group and other groups (Wang et al., 2014; Zhang et al., 2017).

In our study, we identified 5381 DE target mRNAs for the *cis*-regulated target genes of the DE lncRNAs. To assess the key factors involved in the regulation of biological pathways, these 5381 target mRNAs were further analyzed, and nine enriched KEGG pathways were identified, including the Hippo signaling pathway, cell cycle, carbon metabolism, and fatty acid metabolism (Supplementary Table S6). First, we discovered that the Hippo signaling pathway was the most significantly enriched pathway in the KEGG analysis, with 10 DE target genes (Supplementary Table S6). Previous reports have also shown that the Hippo pathway is associated with a broad spectrum of development and stress responses in eukaryotes (Halder and Johnson, 2011; Rock et al., 2013; Di Cara et al., 2015; Mao et al., 2015; Zheng et al., 2017; Radchenko et al., 2018). For example, Di Cara et al. (2015) found that the Hippo pathway promotes cell survival in response to chemical stress. In addition, Zheng et al. (2017) have reported that the lncRNAs associated with glycolysis were upregulated in response to glucose stimulation treatment and are most likely responsible for energy stress. Here, we found that several target genes (such as XM_007821819.1 and XM_007826591.1) that are involved in the Hippo signaling pathway were upregulated under heat stress conditions, indicating that this pathway plays significant roles in the response to heat stress. More importantly, one protein kinase gene (serine/threonine protein kinase, XM_007821819.1) that was upregulated under heat stress conditions was also found to be shared in this pathway and the cell cycle (the second most enriched pathway in the KEGG analysis). Second, another significantly enriched pathway is the cell cycle (Supplementary Table S6), as indicated by the KEGG analysis. It has previously been reported that to maximize survival, cells delay the progression of the cell cycle in response to environmental stress (Sole et al., 2015). Furthermore, recent studies have shown that lncRNAs are involved in the regulation of cell cycle progression during adaptation to conditions of stress (Nadal-Ribelles et al., 2014). For instance, Cdc28 (a cyclin-dependent kinase), which modulates the cell cycle in yeast, was governed by one stress-induced lncRNA (Nadal-Ribelles et al., 2014; Sole et al., 2015). Indeed, Cdc28 lncRNA mediates the induction of *CDC28* expression, and the increased expression level of Cdc28 ensures the effectivity of cellular re-entry into the cell cycle after osmotic stress (Nadal-Ribelles et al., 2014; Sole et al., 2015). In our analysis, dozens of target genes participated in the cell cycle (Figure 4B), showing differential expression under heat stress conditions. Combined with the analysis of the Hippo pathway mentioned above, we found that several stress-related lncRNAs and their target genes (including LTCONS_00014040, LTCONS_00003164, XM_007821819.1, XM_007821219.2, XM_007822193.1, and XM_007821296.2) were shared among the Hippo pathway and cell cycle. In fact, several previously published reports have shown that the cell cycle in yeast can, at least in part, be regulated by the Hippo signaling pathway (Hsu and Weiss, 2013; Rock et al., 2013). In addition, stress can cause a decrease in related kinase activity and a cell cycle delay in *Saccharomyces cerevisiae* (Belli et al., 2001). Therefore, it seems meaningful to focus on these shared lncRNAs and signaling pathways, as these overlapping signaling pathways are likely to play significant roles in the heat stress response in *M. robertsii*.

In addition, among the target genes of lncRNAs, two classic genes that may be involved in the heat stress response were identified (Supplementary Table S5). For example, the hsp70-like gene (XM_007821830.1), which encodes heat shock protein 70, was a putative target of the adjacent lncRNA LTCONS_00010017. In particular, the expression of the hsp70-like gene and LTCONS_00010017 increased markedly under heat stress conditions. The same result was observed for another hsp 70 (XM_007825705.1) and lncRNA (LTCONS_00000109). These results were largely consistent with those of previous studies by our group and other groups, that is, heat shock proteins and other chaperones are selectively expressed in response to heat shock (Parsell and Lindquist, 1993; Lakhotia, 2012; Wang et al., 2014; Valadkhan and Valencia-Hipolito, 2016).

In conclusion, many lncRNAs involved in the heat stress response in *M. robertsii* were discovered and characterized, and their potential *cis*- and *trans*-acting functions were predicted based on their corresponding DE target mRNAs. These results provide a basis for the lncRNAs produced in *M. robertsii* in response to heat stress and expand our knowledge regarding the lncRNAs involved in the heat stress response in filamentous fungi.

## Data Availability Statement

The datasets generated for this study can be accessed from the National Center for Biotechnology Information (NCBI) database under the accession number PRJNA232660.

## Author Contributions

BH and ZW conceived and designed the study. ZW and YJ wrote the manuscript and analyzed the data. YJ, HW, and XX conducted a part of the experiments and data analysis. BH edited the manuscript and supervised the project. All authors contributed to the manuscript revision, and read and approved the submitted version.

## Conflict of Interest

The authors declare that the research was conducted in the absence of any commercial or financial relationships that could be construed as a potential conflict of interest.
