# Supplementary material for: Genome-Wide Identification and Functional Prediction of Long Non-coding RNAs Involved in the Heat Stress Response in Metarhizium robertsii
Source: Front Microbiol. 2019 Oct 9;10:2336. doi: 10.3389/fmicb.2019.02336 (PMC6794563; doi:10.3389/fmicb.2019.02336)
Supplement: Supplementary file 1 [file Data_Sheet_1.zip › Data Sheet 1/Supplementary Materials/Figure S1-3 and Table S8.doc]

Supplementary Materials for

**Genome-wide identification and functional prediction of long noncoding RNAs involved in the heat stress response in *Metarhizium robertsii***

Zhangxun Wang^1, 2, †^, Yuanyuan Jiang^1, 2, †^, Hao Wu^1^, Xiangyun Xie^1^ and Bo Huang ^1,*^

^1^ Anhui Provincial Key Laboratory of Microbial Pest Control, Anhui Agricultural University, Hefei 230036, China

^2^ School of Plant Protection, Anhui Agricultural University, Hefei 230036, China

^†^ These authors contribute equally to this study.

^*^ Corresponding author.

Anhui Provincial Key Laboratory of Microbial Pest Control, Anhui Agricultural University, Hefei 230036, China.

Tel./ Fax: +86-551- 65786211.

Email: bhuang@ahau.edu.cn

**FIGURE S1. Gene deletion and complementation** **of *mrLncRNA1* in *M. robertsii*.**

**(a)** Schematic diagram of *mrLncRNA1* disruption by a homologous recombination approach. A 726-base-pair fragment of the *mrLncRNA1* coding region was replaced with the 942-base-pair *bar* cassette through homologous recombination between the overlapping regions of the 5′ and 3′ fragments in *mrLncRNA1* and p*bar*-*mrLncRNA1* (recombinant vector), respectively. The corresponding locations of the primers are indicated by arrows.

**(b)** PCR confirmation. Genomic DNAs extracted from different strains were used as templates for PCR. The upstream fragment, the downstream fragment, *mrLncRNA1*, *bar*, and *ben* were amplified with the primer sets P5/P6, P7/P8, P1/P2, P3/P4, and *ben*-F/*ben*-R, respectively (see Table S1). Initially, two recombinant fragments of 1692 and 1683 bp were only amplified with the primer sets P5/P6 and P7/P8 in the mutant Δ*mrLncRNA1* but not in WT. The *mrLncRNA1* fragment (132 bp) was amplified with the primer set P1/P2 from WT and Comp but not from ∆*mrLncRNA1*. Part (434 bp) of the *bar* (glufosinate ammonium resistance) fragment was amplified with the primer set P3/P4 from Δ*mrLncRNA1* and Comp mutants but not from WT. Additionally, the benomyl resistance gene fragment (*ben*, 301 bp) was amplified with the primer set *ben*-F/*ben*-R from Comp but not from WT and Δ*mrLncRNA1*. M: marker; 1: WT (wild-type strain), 2: MT (Δ*mrLncRNA1* strain), 3: Comp (complemented strain).

**(c)** RT-PCR verification of the gene expression of *mrLncRNA1* in the WT, Δ*mrLncRNA1*, and Comp strains. Using cDNA as a template, *mrLncRNA1* was amplified with the primer set P1/P2 from WT and Comp but not fromΔ*mrLncRNA1*; transcripts of *mrLncRNA1* were completely undetectable in the gene deletion mutant and were detected in the WT and Comp strains. The expression level of glyceraldehyde 3-phosphate dehydrogenase (*gpd*) (MAA_07675) was used as an internal control.

**FIGURE S2. Heatmap of the expression analysis for lncRNAs and their *trans*-regulated target genes.**

**FIGURE S3. KEGG pathway distributions of 7003 differentially expressed genes in the heat-treated and control conidia samples.**

A circle represents the corresponding target genes enriched in the pathway, and the size is proportional to the number of gene. The *Y*-axis shows the corresponding enrichment pathway. The *X*-axis indicates the enrichment factor.

**Table S8. LncRNAs identified in fungi**

| **No.** | **Fungus** | **Features of genomic locations of lncRNAs** | | | | **Function in *cis/trans*** | **Reference** |
| --- | --- | --- | --- | --- | --- | --- | --- |
|  |  | Intergenic lncRNAs  (LincRNAs) | Anti-sense lncRNAs | Sense lncRNAs | Intronic lncRNAs |  |  |
| 1 | *Metarhizium robertsii* | 1416 | 277 | 42 | 7 | *Cis/trans* | ***This study*** |
| 2 | *Neurospora crassa* | 1478 | 1056 | - ^a^ | - | ? ^b^ | **Cemel et al., 2017** |
| 3 | *Coniophora puteana* | 2059 | 607 | - | 46 | *Cis* | **Borgognone et al., 2019** |
| 4 | *Serpula lacrymans* | 1912 | 314 | - | 16 | *Cis* | **Borgognone et al., 2019** |
| 5 | *Phytophthora sojae* | 898 | 73 | 35 | 4 | *Cis* | **Wang et al., 2018** |
| 6 | *Fusarium graminearum* | 237 | 280 | - | - | *Cis* | **Kim et al., 2018** |
| 7 | *Cordyceps militaris* | 4140 ^c^ | - | - | - | *Cis* | **Wang et al., 2019** |
| 8 | *Pichia pastoris* | 36 | 168 | - | 4 | *Cis/trans* | **Sun et al., 2019** |

**Note:**

^a^ It is not described in the corresponding references.

^b^ It is unclear. *cis*-regulatory lncRNAs mainly control the expression of neighboring genes (close genomic proximity), and *trans*-regulatory lncRNAs modulate the expression of distant genes.

^c^ There is no detail in the corresponding reference.

**References**

Borgognone, A., Sanseverino, W., Cigliano, R.A., and Castanera, R. (2019). Distribution, characteristics, and regulatory potential of long noncoding RNAs in brown-rot fungi. *International Journal of Genomics***,** 9702342. doi: 10.1155/2019/9702342.

Cemel, I.A., Ha, N., Schermann, G., Yonekawa, S., and Brunner, M. (2017). The coding and noncoding transcriptome of *Neurospora crassa*. *BMC Genomics* 18(1)**,** 978. doi: 10.1186/s12864-017-4360-8.

Kim, W., Miguel-Rojas, C., Wang, J., Townsend, J.P., and Trail, F. (2018). Developmental dynamics of long noncoding RNA expression during sexual fruiting body formation in *Fusarium graminearum*. *mBio* 9(4)**,** e01292-01218. doi: 10.1128/mBio.01292-18.

Sun, W., Wang, Y., Xu, Y., and Yu, X. (2019). Genome-wide analysis of long non-coding RNAs in *Pichia pastoris* during stress by RNA sequencing. *Genomics* 111(3)**,** 398-406. doi: 10.1016/j.ygeno.2018.02.016.

Wang, Y., Shao, Y., Zhu, Y., Wang, K., Ma, B., Zhou, Q., et al. (2019). XRN1-associated long non-coding RNAs may contribute to fungal virulence and sexual development in entomopathogenic fungus *Cordyceps militaris*. *Pest Management Science*. doi: 10.1002/ps.5453.

Wang, Y., Ye, W.W., and Wang, Y.C. (2018). Genome-wide identification of long non-coding RNAs suggests a potential association with effector gene transcription in *Phytophthora sojae*. *Molecular Plant Pathology* 19(9)**,** 2177-2186. doi: 10.1111/mpp.12692.
